# Supplementary material for: Origins of the hydrothermal dolomites in Middle Permian, Sichuan Basin (SW China): Implication for the relationship with the Emeishan Large Igneous Province
Source: PLoS One. 2025 Nov 20;20(11):e0336625. doi: 10.1371/journal.pone.0336625 (PMC12633893; doi:10.1371/journal.pone.0336625)
Supplement: Attachment — Submitted filename: Reviewer Recommendation.docx [file pone.0336625.s003.docx]

**Reviewer Recommendation: Accept with Minor Revisions**

**Summary of Manuscript**

This manuscript presents a detailed petrographic and geochemical study of Middle Permian dolomites (specifically types Rd3 and Sd) from the Sichuan Basin, China. The authors aim to determine the origin of these dolomites and establish their relationship with the Emeishan Large Igneous Province (ELIP). Through an integrated analysis of core, outcrop, petrography, fluid inclusion microthermometry, and stable (C, O) and radiogenic (Sr) isotopes, the study concludes that the formation of these dolomites was directly controlled by the ELIP.

This is a well-researched and well-written paper that makes a valuable contribution to the understanding of large-scale dolomitization processes linked to igneous activity. The dataset is comprehensive, and the conclusions are well-supported by the evidence.

**General Comments**

The authors have done an excellent job of integrating multiple datasets to build a convincing and regionally significant geological model. The research question is clear, the methodology is appropriate, and the results are presented logically. The link established between the ELIP, fault systems, and the resulting spatial patterns of dolomitization is a key strength of the manuscript. The paper is well-suited for publication and will be of interest to carbonate sedimentologists, petrologists, and economic geologists working on hydrocarbon reservoirs. The following suggestions are intended to further improve the clarity and impact of the manuscript.

**Specific Suggestions for Minor Revisions**

The manuscript is in good shape, and only minor revisions are recommended to enhance its quality. The suggestions are primarily focused on improving the clarity of figures and strengthening minor points in the text.

1. Figure 1: This is a good overview figure. Consider increasing the font size of the labels within Figure 1c (well/outcrop names) for better legibility, especially if the figure is reduced in size for publication.

2. Figure 6 (δ 13C vs. δ18O Plot): This plot is quite busy, and the overlapping dashed boxes for the different regions make it difficult to distinguish the data clusters. To improve clarity, I suggest using a combination of different symbol shapes and colors for each dataset (e.g., squares for SW, circles for Central, triangles for NW). This would make the regional trends you describe in the text much easier for the reader to see visually.

3. Figure 8 (Conceptual Model): This is an excellent summary model. To make it even more effective, consider adding a few more labels to explicitly illustrate the processes. For example:

4. Add arrows and a label for "Heated seawater circulation" in the Northwestern section to visually represent the thermal convection model.

5.Add a label for "Upwelling of deep hydrothermal fluids along faults" in the Southwestern and Central sections.

6. The abstract is well-written. A minor suggestion is to explicitly state that the basement faults acted as the primary fluid conduits, as this is a key part of the model.

7. Methods Section: The analytical methods are standard and well-described. It would be helpful to state the "critical roughening temperature" (CRT) concept briefly in the introduction or discussion when first mentioning non-planar and saddle dolomites, as it is a cornerstone for interpreting their formation temperature.

8. Discussion Section 5.3: In the first paragraph of this section, you state that the high temperature and salinity in areas near faults may be the "result of mixing connate seawater with the hydrothermal fluids." This is a plausible interpretation. Could you briefly elaborate on why this mixing would increase salinity compared to the original hydrothermal fluid, or clarify if the connate water itself was hypersaline? A short sentence of explanation would strengthen this point.

These are all minor points that can be easily addressed by the authors. Once revised, this paper will represent a strong and impactful contribution to the field.
