# Supplementary material for: Origins of the hydrothermal dolomites in Middle Permian, Sichuan Basin (SW China): Implication for the relationship with the Emeishan Large Igneous Province
Source: PLoS One. 2025 Nov 20;20(11):e0336625. doi: 10.1371/journal.pone.0336625 (PMC12633893; doi:10.1371/journal.pone.0336625)
Supplement: Attachment — Submitted filename: Response to Reviewers.docx [file pone.0336625.s005.docx]

**Manuscript Title:** Origins of the hydrothermal dolomites in Middle Permian, Sichuan Basin (SW China): Implication for the relationship with the Emeishan Large Igneous Province

**Authors:** Haofu Zheng, Jiajun He, Xiong Duan, Bo Liu, Anna Travé Herrero, Juan Diego Martín-Martín, Enrique Gómez Rivas

Dear Editor,

Please find attached our revised manuscript (clean and tracked-changes versions) and the point-by-point response to the reviewers.

Below we confirm that the editorial and ethical requirements have been fully addressed.

1. Formatting. We have carefully adjusted the entire manuscript to conform to PLOS ONE style as requested. If any further formatting details are still required we will correct them immediately.

2. Ethics and permissions. Fieldwork did not involve endangered species, protected areas, or sensitive/private information; therefore no additional institutional permissions were necessary.

Core observation and sampling were carried out under the written permission granted by the Exploration Branch of Sinopec.

3. Funding. All grant numbers and funding bodies have been checked and verified against the original award letters.

4. Data availability. All data needed to evaluate the conclusions are contained within the paper and the supplementary file “Appendix of the manuscript.xlsx”.

5. Acknowledgements. Funding-related text has been removed from the Acknowledgements section and is now reported exclusively in the “Funding” section. The Acknowledgements have been rewritten accordingly.

6. Copyright.

• Figure 1: the base map is cited to its original open-access source and therefore no copyright issues are anticipated.

• Figure 3: contains no third-party map material and is free of copyright concerns.

We thank you and the reviewers for your constructive comments, which have substantially improved the clarity and rigour of our work.

Sincerely,

Xiong Duan

***Response to Reviewers***

We would like to extend our sincerest gratitude to the academic editor and both reviewers for their time, effort, and insightful feedback on our manuscript. The comments are highly constructive and have significantly improved the quality and clarity of our work. We have carefully considered all suggestions and have revised the manuscript accordingly. We are also grateful for the opportunity to incorporate several very recent and relevant studies, which have further strengthened our interpretations. Below, we provide a point-by-point response to each of the reviewers' comments, detailing the changes we have made.

***Response to Reviewer #1 (Dr. Cole A. McCormick)***

We are particularly grateful to Dr. McCormick for the exceptionally thorough and expert review. Your detailed suggestions, deep knowledge of hydrothermal systems, and provision of relevant literature have been invaluable in strengthening our manuscript's arguments. The additional literature we have incorporated further supports the models discussed and addresses the complexities you highlighted. We have addressed each of your points as detailed below.

Reviewer Comment #1: Need to define “hydrothermal” dolomite at the beginning of the Introduction. I would suggest using the definition from Machel and Lonnee (2002). Remove citations to Smith and Davies (2006) and Davies and Smith (2007) unless there are specific details from these papers that you want to present. Note that it is not always the case that HTD forms in “shallow-buried” strata.

Response: We thank Dr. McCormick for this excellent and foundational suggestion. We agree that a clear and rigorous definition of "hydrothermal" is essential and that the definition provided by Machel and Lonnee (2002) is the most appropriate standard for the field. We have rewritten the first paragraph of the Introduction to incorporate this definition and have removed the generalization about HTD forming only in "shallow-buried" strata. Our citations are now more focused and relevant.

Reviewer Comment #2: I really like how the authors present a clear list (i to iv) at the end of the Introduction stating their research goals and objectives. However, a manuscript needs to present more than just a documentation of these aspects. What broader scientific problem are you trying to address, why has this not been studied before or why were previous studies insufficient, and what are the broader implications of your work?

Response: This is a crucial point, and we thank the reviewer for pushing us to better articulate the novelty and broader impact of our study. Our study is novel in its basin-scale comparative approach, systematically linking the geochemical gradients of dolomitizing fluids across the vast Sichuan Basin directly to the proximity of a major tectono-thermal event (the ELIP). Previous studies have been more localized. The addition of new literature, especially recent U-Pb dating studies, allows us to frame our work within a new understanding of multiple, discrete hydrothermal events within the basin, making a basin-scale comparative study timely and necessary.

We have revised the final paragraph of the Introduction to better frame our contribution.

Reviewer Comment #3: The authors use “replacive dolomite” and “dolomite cement” as their petrographical classifications... Why not just call this ‘non-planar, saddle dolomite’ in the abstract, and use the abbreviation ‘Sd’ as you do throughout the manuscript? Please simply use the term “zebra texture” in Line 170 and in the figure caption for Fig. 3.

Response: We completely agree with this comment. Using descriptive terms in observational sections is more rigorous. We have revised the manuscript to use "saddle dolomite (Sd)" instead of "dolomite cement (Sd)" in the Abstract and Results sections. The interpretation of Sd as a cement is now confined to the Discussion. We have also corrected the terminology to "zebra texture" as requested.

Reviewer Comment #4: If there are any leftover powders, it would be excellent to include some XRD data in this manuscript... For future studies, I would strongly suggest that the authors conduct XRD on all their powders before geochemical analyses.

Response: We thank the reviewer for this practical suggestion. We agree that XRD analysis is a standard and valuable tool. Unfortunately, due to the consumption of sample powders, we do not have sufficient material remaining for new XRD analyses.

Reviewer Comment #5: The authors do an excellent job of presenting their petrographical and geochemical results... Well done!

Response: We thank the reviewer for their positive feedback.

Reviewer Comment #6: The authors have collected both d18O ratios and fluid inclusion homogenization temperatures from the same samples. Clearly, they also need to calculate the d18O values of the diagenetic fluid! I would like to see a plot of the d18O(fluid) values for each of these locations as well. I would suggest using Horita (2014).

Response: This is an excellent point. We have now calculated the δ¹⁸Ofluid values using our mean Th data and dolomite δ¹⁸O values, applying the dolomite-water fractionation equation from Horita (2014). We have created a new figure to display these results and have incorporated this new data into our discussion, which greatly strengthens our interpretations.

Reviewer Comment #7: I would like to see the authors present a conventional “paragenetic sequence” type diagram as an added figure.

Response: This is another excellent suggestion. We have created a new figure that illustrates the relative timing of all observed diagenetic phases against the burial history of the basin (Fig.10).

Reviewer Comment #8: A major criticism I have is that the Discussion is quite limited... this needs to be much much more thorough, with further discussion of how these processes operate within the Sichuan Basin and then with a clear discussion of other sedimentary basins worldwide.

Response: We fully agree with this criticism and thank the reviewer for pushing us to significantly improve this section. We have substantially rewritten and expanded the Discussion section, incorporating the new literature you and we have found. This has allowed us to present a more sophisticated model that considers concepts like fluid- vs. rock-buffered systems and places our specific Permian event within the context of multiple hydrothermal pulses in the basin's history.

The Discussion has been restructured and expanded as follows:

(8a) Fluid Mixing/Continuum & Rock- vs. Fluid-Buffering: We have revised the "Dolomitization mechanisms" section to present a more nuanced model, now framed with the concepts of fluid- and rock-buffered systems, drawing on the work of Jiang et al. (2025).

(8b) Comparison to other strata in the Sichuan Basin: We have added a new discussion in 5.3 to place our findings in the context of a multi-stage hydrothermal history, citing the crucial U-Pb dating studies by Li et al. (2023) and Zou et al. (2023). This directly addresses the complex interplay of the ELIP and the Longmenshan Orogeny as drivers for fluid flow.

(8c) Global Context: We have expanded this section, comparing our findings to the some other sedimentary basins all over the world.

***Response to Reviewer #2***

We thank Reviewer #2 for their valuable comments, which have helped us improve the clarity and presentation of our manuscript. We have addressed each suggestion below.

Reviewer #2, Comment (1, 7): Figure 1: ...increasing the font size of the labels... Figure 1c...

Our Response: We agree that the labels were too small.

Action Taken: The font size of all labels in Figure 1c has been increased to ensure legibility, even if the figure is reduced in size for publication.

Reviewer #2, Comment (2, 10): Figure 6 (δ13C vs. δ18O Plot): This plot is quite busy... I suggest using a combination of different symbol shapes and colors for each dataset...

Our Response: I think we've made the legend clear, using shapes to distinguish between minerals and colors to distinguish between regions.

Reviewer #2, Comment (3-5): Figure 8 (Conceptual Model): ...consider adding a few more labels... "Heated seawater circulation"... "Upwelling of deep hydrothermal fluids along faults"...

Our Response: We agree that adding these labels would make our conceptual model more explicit and effective.

Action Taken: We have added arrows and labels to Figure 8 to explicitly illustrate "Heated seawater circulation" in the Northwestern section and "Upwelling of deep hydrothermal fluids along faults" in the Southwestern and Central sections, as suggested .

Reviewer #2, Comment (6): The abstract is well-written. A minor suggestion is to explicitly state that the basement faults acted as the primary fluid conduits...

Our Response: We agree this is a key point that should be highlighted in the abstract.

Action Taken: We have added a sentence to the abstract to explicitly state that deep-seated basement faults, reactivated during the ELIP event, acted as the primary conduits for hydrothermal fluid migration.

Reviewer #2, Comment (7): Methods Section: It would be helpful to state the "critical roughening temperature" (CRT) concept briefly... when first mentioning non-planar and saddle dolomites...

Our Response: This is a helpful suggestion to provide necessary background for the reader.

Action Taken: We have added a brief explanation of the "critical roughening temperature" (CRT) concept from Sibley and Gregg (1984) in the Discussion (Section 5.1) where we first interpret the significance of the non-planar crystal textures.

Reviewer #2, Comment (8): Discussion Section 5.3: ...Could you briefly elaborate on why this mixing would increase salinity compared to the original hydrothermal fluid, or clarify if the connate water itself was hypersaline?

Our Response: There may be an error in the original manuscript, this mixing does not increase the salinity of the original hydrothermal fluid. We have rewritten Discussion 5.3 to avoid this erroneous statement.

We believe that the manuscript has been significantly strengthened by incorporating the reviewers' feedback and the latest relevant literature. The discussion is now more robust, the context within the Sichuan Basin is clearer, and the global relevance of our findings is better articulated. We hope that our revisions are satisfactory and that the manuscript is now suitable for publication.

Sincerely,

Haofu Zheng and co-authors.
